# Peening Techniques for Mitigating Chlorine-Induced Stress Corrosion Cracking of Dry Storage Canisters for Nuclear Applications

**DOI:** 10.3390/ma18020438

**Published:** 2025-01-18

**Authors:** Subin Antony Jose, Merbin John, Manoranjan Misra, Pradeep L. Menezes

**Affiliations:** 1 Department of Mechanical Engineering, University of Nevada, Reno, NV 89557, USA; subinj@unr.edu (S.A.J.); merbinjohn.2011@gmail.com (M.J.); 2 Department of Chemical and Materials Engineering, University of Nevada, Reno, NV 89557, USA; misra@unr.edu

**Keywords:** spent nuclear fuel, chloride-induced stress corrosion cracking, shot peening, laser shock peening, hybrid welding

## Abstract

Fusion-welded austenitic stainless steel (ASS) was predominantly employed to manufacture dry storage canisters (DSCs) for the storage applications of spent nuclear fuel (SNF). However, the ASS weld joints are prone to chloride-induced stress corrosion cracking (CISCC), a critical safety issue in the nuclear industry. DSCs were exposed to a chloride-rich environment during storage, creating CISCC precursors. The CISCC failure leads to nuclear radiation leakage. Therefore, there is a critical need to enhance the CISCC resistance of DSC weld joints using promising repair techniques. This review article encapsulates the current state-of-the-art of peening techniques for mitigating the CISCC in DSCs. More specifically, conventional shot peening (CSP), ultrasonic impact peening (UIP), and laser shock peening (LSP) were elucidated with a focus on CISCC mitigation. The underlying mechanism of CISCC mitigation in each process was summarized. Finally, this review provides recent advances in surface modification techniques, repair techniques, and developments in welding techniques for CISCC mitigation in DSCs.

## 1. Introduction

**Figure 1:**
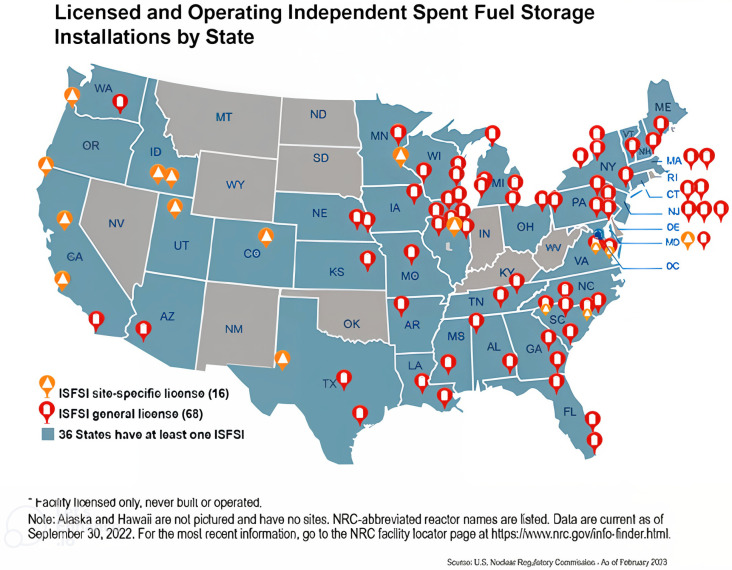
Locations of the US licensed and operating independent fuel storage installations. Reproduced with permission from [2].

Currently, the United States (US) possesses 80,000 metric tons of spent nuclear fuels (SNFs) in approximately 1500 dry storage canisters (DSCs) at 63 independent storage locations [1], made from welded austenitic stainless steel (ASS). The expected storage duration of SNFs in DSCs is beyond 60 years. Figure 1 shows the licensed and independent storage locations of the DSCs across the US. The US Energy Information Administration (EIA) report shows that SNF storage readily increased from 2013 to 2017. Between 1968 and 2017, more than 276,000 bundles of SNFs were stored in interim storage locations.

**Figure 2:**
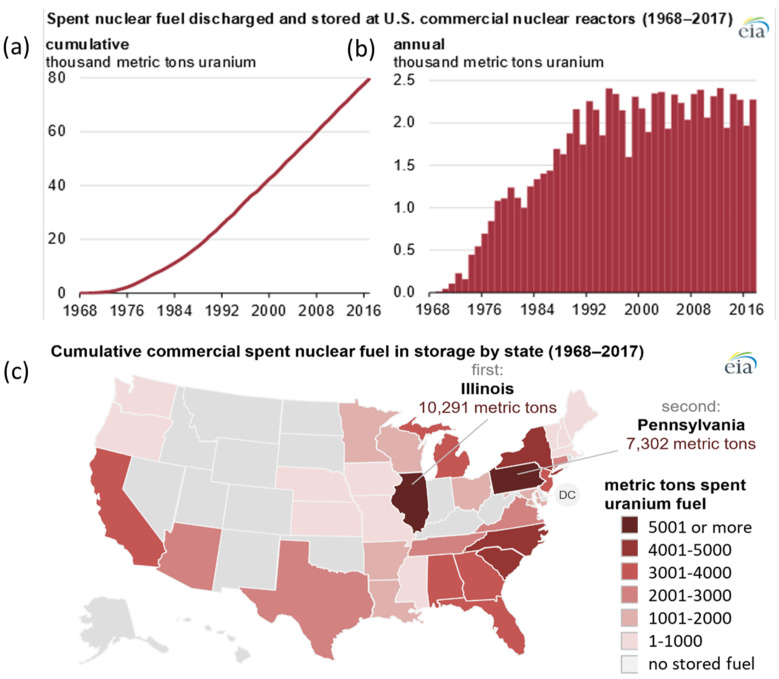
SNF storage at interim locations in metric tons of uranium (**a**) cumulative, (**b**) annual, and (**c**) state wise [3].

Figure 2a,b shows the cumulative SNF and annual SNF storage at the interim storage location. The data demonstrate that the volume of SNFs generated from the nuclear power plants in the US has continuously increased in recent decades. The cumulative storage of SNFs between 1968 and 2017 corresponding to each US states is shown in Figure 2c. These data report that Illinois and Pennsylvania store more than 175,000 metric tons of SNFs. During storage, DSCs exposed to a chloride-rich environment experience the formation of chloride-laden deliquescent brines on their surfaces, leading to chloride-induced stress corrosion cracking (CISCC).

The CISCC can be intergranular and transgranular. The intergranular CISCC can be controlled by choosing lower carbon compositions. The transgranular CISCC is still a nightmare in nuclear applications [4]. CISCC failure eventually causes nuclear radiation leakage and affects the populations residing near the interim storage locations. The CISCC occurs in the ASS weld joints of DSCs because of the synergistic effect of susceptible material, in the presence of corrosive environment and residual tensile stress (RTS) [5]. Joining of ASS for DSCs is generally carried out by gas metal arc welding (GMAW). The melting and solidification during welding induce significant RTS and large heat-affected zone (HAZ) that affects the weld joint quality and integrity. The larger heat input and the melting followed by rapid cooling during the GMAW process result in a coarse, segregated microstructure that is prone to CISCC. Heat treatment is not applied to these joints after welding to relieve the RTS. It develops chromium-depleted zones in grain boundaries in the HAZ, leading to intergranular CISCC [6,7,8]. Therefore, there is a critical need to enhance the CISCC resistance of DSCs for storing SNFs. The gap analysis performed by the Department of Energy (DoE) reported that GMAW joints were sensitive to CISCC [9]. As a result, it is of utmost importance to manufacture corrosion-resistant weld joints to provide sustained integrity and reliability and comply with SNF storage regulatory standards. Previously, GMAW has also been explored as a repair method to address CISCC in fusion-welded regions of DSCs. However, this repair technique incorporates melting and solidification, which can potentially lead to the formation of segregated microstructures and the development of shrinkage stresses in the freshly formed HAZ [10]. Moreover, processes with such higher heat inputs are generally undesirable for the safety of on-site canister manufacture and repair. The CISCC can significantly affect the safety of the DSC and can lead to the formation of partial or through wall cracks in the DSC. The cracks detected in the Koeberg and Turkey Point nuclear power stations are considered potential CISCC issues [11]. Controlling RTS on the weld joint is the only way to prevent the CISCC failures of DSC weld joints. The material or environment cannot be changed for SNF storage application [12].

Scholars introduced different severe plastic deformation (SPD) techniques to prevent CISCC in DSCs [13]. These techniques were predominantly used to prevent failures occurring from the surface, such as wear, corrosion, and fatigue [14]. Among the different SPD methods, peening techniques are commonly employed as a mechanical surface treatment, which is simple, robust, and the most effective top-down industrially viable technique [15,16]. These techniques induce grain refinement, enhance surface mechanical properties, and change the tensile stress to compressive. These techniques can be categorized as conventional shot peening (CSP), ultrasonic impact peening (UIP) [17], and laser shock peening (LSP) [18]. There are many controllable parameters associated with these techniques. These techniques help to prevent crack nucleation, existing crack growth, and propagation. Among these techniques, CSP was developed in the early 1950s as a repair method aimed at improving the surface mechanical properties of aerospace components. The CSP induces grain refinement, phase transformations, residual compressive stress (RCS) [19,20], and surface integrity changes [16,17]. Later, advanced forms of CSP, such as severe shot peeing (SSP) and micro-shot peening (MSP), were developed [21,22,23]. These techniques could produce superior surface properties compared to CSP and have been applied to many engineering materials [22,23,24]. Later, at the beginning of the 1960s, high-frequency ultrasonic vibrations were employed to enhance the surface properties of engineering materials [25,26]. This involves subjecting the substrate material to an ultrasonic frequency using a tooltip that induces RCS, grain refinement, and plastic deformation [27,28]. The plastically deformed layer formed after UIP treatment possesses superior properties compared to the bulk of the specimen. However, to address adverse and potential problems due to corrosion, wear, and fatigue, laser-based surface engineering techniques emerged in the late 1960s and early 1970s [29,30]. These techniques could develop superior properties on engineering materials and prevent many setbacks associated with CSP and UIP. LSP is the predominant method of laser-based surface modification. This process involves the interaction of lasers with high energy density with a substrate material in a confinement medium [31]. The plastic deformation resulting from LSP can improve surface properties, including hardness, wear resistance, CISCC resistance, and fatigue strength [32,33,34].

This review paper offers an in-depth examination of peening techniques for mitigating CISCC issues in DSCs. Section 2 delve into the CISCC mechanisms in DSC weld joints. Section 3 offers an in-depth analysis of peening techniques, with a specific focus on CSP, UIP, and LSP methods. While Section 4 discusses various testing methods for evaluating SCC in ASSs, Section 5 examines the factors influencing SCC resistance in materials. Lastly, recent advancements in mitigating CISCC in DSC weld joints are explored in Section 6.

## 2. CISCC Mechanism

The CISCC research was widely focused on diverse industries over many decades. However, none of the research discussed the exact mechanism. This is due to the dependence of CISCC on the environmental condition and type of materials under consideration. In a chloride-rich environment, the CISCC mechanism involves two steps. The mechanism includes an initiation phase, followed by a crack propagation phase. In the initiation stage, electrochemical mechanism dominates. This stage comprises the pit initiation. Pits act as the precursor to the CISCC. In the propagation stage, electrochemistry and metal separation dominate. Here, the material degradation takes place.

### 2.1. The Initiation Stage

**Figure 3:**
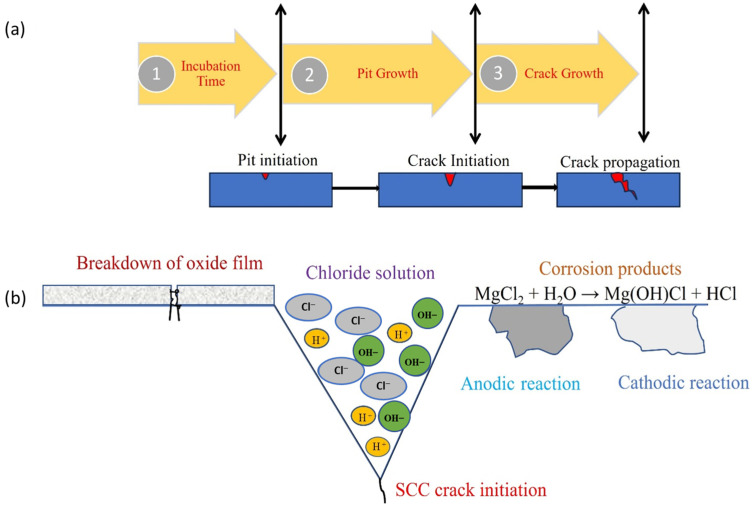
Mechanism of CISCC process: (**a**) stages depicting the progression from pit initiation to crack propagation; (**b**) close-up view highlighting the initiation of SCC. Reproduced with permission from [39].

As mentioned, stainless steel possesses superior generalized corrosion resistance. This is primarily because of the formation of the Cr_2_O_3_ passive layer [35]. However, it is highly susceptible to an aggressive chloride environment. In an aggressive environment, pits initiate. Generally, it initiates on inclusions, solute-segregated grain boundaries, heterogeneities, and mechanically damaged locations. Additionally, there may be a highly susceptible site where pits initially form. Under aggressive chloride ions, the passive layer on the stainless steel is locally damaged. The potential at which the material loses passivity is referred as pitting potential or film breakdown potential [36]. The pitting corrosion generally depends on the concentration of chloride ions. The pitting generally consists of two stages based on the progress of the initiated pits. It can be of stable pitting or metastable pitting. Pits nucleate when aggressive species break and penetrate the passive film [37]. More specifically, the progression of CISCC can be categorized by incubation time, pit growth, and crack growth [38] (Figure 3a). Incubation time is the time during which no pits initiate. After the incubation period, pits begin to form on the surface. This is where the passive layer has been compromised. Initially, small pits nucleate and remain in a metastable state.

During the pit growth stage, cracks initiate at the pit sites, while in the subsequent crack growth stage, these cracks propagate deeper into the bulk material along the thickness direction. Figure 3b illustrates the macroscopic process of crack initiation. In this process, pits on the damaged layer act as anodes, whereas the regions covered by the passive layer serve as cathodes. The anodic and cathodic reactions occurring in boiling MgCl_2_, a highly aggressive chloride solution, are represented by Equations (1)–(3) [40].

(1) $$\mathrm{Anode}:M\to M^{n+}+\mathrm{ne}$$

(2) $$\mathrm{Cathode}:{\mathrm{nFe}}^{3+}+\mathrm{ne}\to {\mathrm{nFe}}^{2+}$$

(3) $${\mathrm{MgCl}}_{2}+H_{2}O\to \mathrm{Mg}\left(\mathrm{OH}\right)\mathrm{Cl}+\mathrm{HCl}$$

### 2.2. The Propagation Stage

Cracks originate at the pits and propagate from the surface into the bulk of the substrate. During this process, multiple cracks may interact. The crack tip develops a negative charge, attracting metal ions toward it. The accumulation of metal ions at the crack tip increases their concentration, ultimately leading to metal dissolution [40]. The rapid progression of CISCCs is driven by the presence of RTS and metal dissolution. As time progresses, both the rate of metal dissolution and the rate of SCC propagation increase [41]. Turnbull et al. discusses the harmful effect of RTS on SCC resistance [42]. Once the CISCC starts, it propagates rapidly. This leads to a situation in which catastrophic failure of the DSCs occurs, and nuclear radiation is released from the canister. This is a critical situation as it can affect the people residing near the interim storage locations. This necessitates and demands the need for post-processing or repair techniques to prevent the setbacks associated with CISCC failure of DSCs.

## 3. Peening Techniques

**Figure 4:**
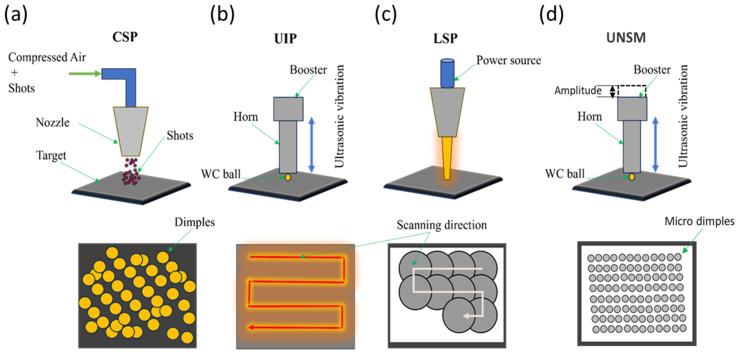
The schematic of the peening process (**a**) CSP, (**b**) UIP (**c**) LSP, and (**d**) UNSM.

Peening techniques were widely employed for enhancing the surface mechanical properties and integrity of various engineering materials for diverse industrial applications. These methods include CSP, UIP, LSP, UNSM (ultrasonic nanocrystal surface modification), and several techniques that involve modifications of these approaches. The selection of a particular method for an application depends on many factors. The schematic illustration of the peening techniques is shown in Figure 4. These processes were discussed in sub-sections of Section 3.

**Figure 5:**
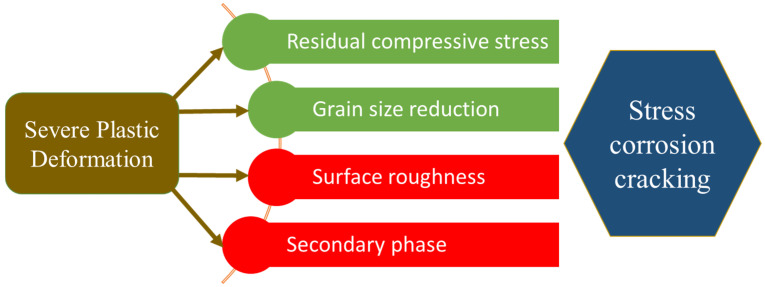
Factors that predominantly influence the CISCC resistance due to the SPD process.

The output parameters of the SPD process can positively or negatively affect the CISCC resistance. The positive factors include RCS and refinement of grains. The synergistic effect of these two factors enhances the CISCC resistance. Refined grains have higher CISCC resistance than their coarse counterparts [17,43]. The evolution of RCS and refined grains can reduce the stress corrosion sensitivity index. Thus, the stress intensity factor reduces, and the RCS in the surface-modified region makes crack propagation difficult [44]. The negative factors include surface roughness and phase transformations [45]. The surface roughness leads to stress concentration and provides conducive crack propagation conditions [46]. The presence of second-phase formation during SPD leads to the selective dissolution of one of the phases and provides an easy path for crack propagation [45,47]. The most influential parameters that oppose/support the CISCC resistance are shown in Figure 5. Hence, careful selection of appropriate parameters during these peening processes is paramount.

The following sub-sections provide a detailed overview of each peening process and its effect on CISCC resistance. The discussion involves four factors mentioned in Figure 5, and how these factors influence the CISCC resistance in each processes.

### 3.1. Conventional and Advanced Shot Peening Techniques

**Figure 6:**
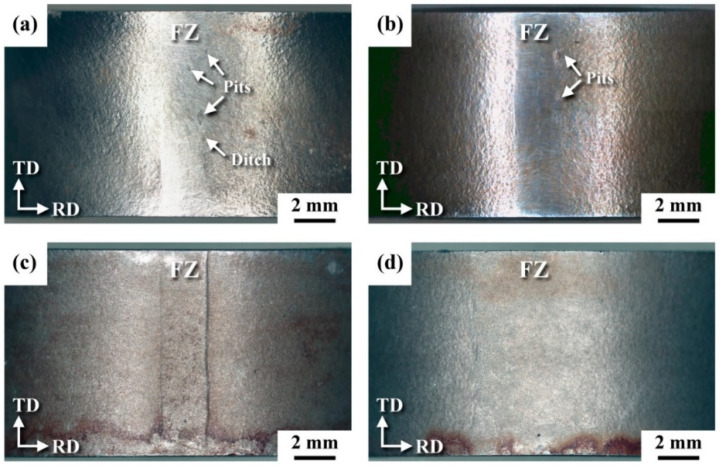
Surface morphology of the (**a**) SS 304 weld, (**b**) SS 316 weld, (**c**) SS 304 weld that underwent MSP, and (**d**) SS 316 weld that underwent MSP. Reproduced with permission from [51].

In the CSP, spherical shots are propelled at controlled velocities toward the substrate material using compressed air through a nozzle. The interaction between the shots and the substrate creates an elastoplastic deformation zone on the surface. During the recovery phase, the substrate undergoes SPD, resulting in the generation of RCS within the material [48]. The shots can be made of metal, ceramic, and glass beads. Again, the selection of shot type and shot size depends on the needs, demands, and application. The parameters associated with shot peening can be categorized as shot parameters, flow parameters, and target material parameters. The peening intensity and peening coverage are the two critical parameters associated with shot peening [49]. Introducing RCS and grain refinement helps in preventing surface failures, such as corrosion and fatigue. Zhiming et al. [45] conducted SSP experiments on ASS 304L weld joints and studied the CISCC resistance of these weld joints using the slow strain rate tensile (SSRT) test in 3.5 wt.% NaCl. The authors varied the shot peening pressure and correlated it with the stress corrosion sensitivity. They identified that 0.4 MPa shot peening pressure provides the minimum stress corrosion sensitivity index. Below and above 0.4 MPa pressure, the stress corrosion sensitivity index is higher, meaning the material is highly susceptible to CISCC failure. The authors identified two parameters that dominate above and below the 0.4 MPa shot peening pressure, which are grain refinement and phase transformation. When shot, peening pressure increased from zero to 0.4 MPa, grain refinement has a predominant role. The stress corrosion sensitivity index decreases to the point where the shot peening pressure reaches 0.4 MPa. Refined grains can prevent the stress concentration of the cracks, and crack initiation can be delayed. In any situation, if a crack is initiated, the propagation of this crack would be delayed by the high number of grain boundaries. However, when the shot peening pressure is more than 0.4 MPa, the phase transformation played a predominant role in the stress corrosion sensitivity index. The formation of strain-induced martensitic increased with shot peening pressure, reducing the austenitic phase fraction [47]. During corrosion testing, strain-induced martensite undergoes selective dissolution. This martensitic transformation occurs at grain boundaries, resulting in localized chromium depletion. The selective dissolution of the martensite forms pathways that facilitate crack initiation and propagation. Okido et al. [50] also showed that CSP treatment can enhance the CISCC resistance of SS 304 specimens. The authors revealed that the presence of RCS helps to prevent the rupturing of the protective oxide film, and thus, the CISCC resistance improves. Kang et al. [51] employed micro-shot peening (MSP) on ASS 304 and 316 weld joints and studied the CISCC resistance. The primary difference between MSP and CSP is the size difference of the shots. The advantage of small shot size is the decrease in surface roughness accompanied by the generation of high RCS to the substrate material compared to CSP. The authors tested U bend specimens made from SS 304 and SS 316 weld joints in a salt spray of 10% NaCl at 80 °C. Formation of nanostructures, strain-induced martensitic transformation, and introduction of RCS in the weld joints were reported. The salt spray experiments showed the formation of many pits on both unpeened welds as shown in Figure 6a,b. However, both the weld joints were free of pits and fine ditches after peening (Figure 6c,d).

**Figure 7:**
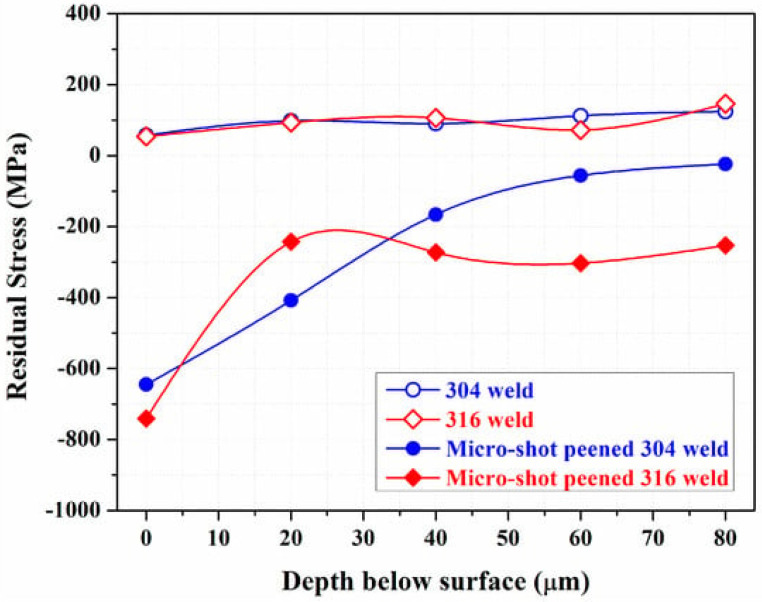
Residual stress distribution in the SS 304 and SS 316 weld joints and weld joints that underwent MSP. Reproduced with permission from [51].

The authors revealed that fine grain formation and RCS prevented the crack initiation and crack propagation of the 304 and 316 weld joints. The residual stress distribution in the 304 and 316 weld and peened weld joints is indicated in Figure 7. It can be visualized from the graph that the weld joint possesses RTS before peening, which is detrimental to CISCC resistance. However, after peening, both the weld joints exhibited RCS. It is observed that RCS is maximum at the surface of the weld joint, and it shows a gradient variation along the depth direction. This demonstrates that the RCS changes to RTS at a particular depth from the surface. The extent to which the RCS can stay within the depth direction depends on the peening parameters, material properties, and material process history [52,53]. The presence of RCS induced on the surface and subsurface regions can effectively prevent crack formation on the surface and hinder its propagation into the subsurface.

Ling et al. [54] conducted CSP experiments on SS 304L weld joints, where they used three coverages; 50%, 100%, and 200%, with glass shots and cast steel shots. The authors revealed that glass shots could induce higher RCS than cast steel shots in all three coverage cases. The CISCC experiments were performed in 42 wt.% boiling MgCl_2_. The unpeened weld joints failed at 6 h, revealing the destructive nature in an aggressive environment. However, the steel shot peened weld joint with 50% coverage withstood 310 h, whereas, a coverage of 100% sustained up to 3500 h of testing. In all the coverage cases with glass bead shots, the weld joints sustained 3500 h of testing. This demonstrates that the selection of the peening parameter, such as coverage and shot material, is paramount in obtaining superior CISCC resistance. These studies summarize that CSP and MSP can effectively eliminate the CISCC failures of DSCs. However, peening parameter selection, especially peening intensity and coverage, are important factors determining how effectively these processes can eliminate CISCC failures. The four parameters, RCS, grain refinement, phase transformation, and surface roughness, significantly affect the CISCC resistance. Ralls et al. [55] used CSP to mitigate SCC in GTAW ASS weld joints with coverages of 100%, 500%, and 1000%. SCC testing on ASS U-bend specimens in boiling MgCl_2_ showed that CSP induced austenite-to-martensite transformation, RCS, and grain refinement, with these effects intensifying as coverage increased. At 100% coverage, SCC resistance significantly improved. However, at 1000% coverage, high martensite content reduced SCC resistance, negating the benefits of RCS and grain refinement. The study also found that CSP changed crack propagation from transgranular to intergranular, delaying crack growth due to the longer path caused by martensite dissolution.

Water Jet Peening is another SPD that uses high-pressure water jets to induce compressive residual stresses on the surface of a material. Unlike traditional shot peening, water jet peening eliminates the need for steel shots, making it particularly advantageous in applications where debris containment is critical, such as in the internal components of pressure vessels in nuclear power plants [56]. This method offers several advantages over traditional shot peening, such as lower stress concentrations, minimal impact on surface roughness, and the ability to effectively treat narrow or intricate parts. Additionally, it is more efficient and environmentally sustainable [48].

### 3.2. Ultrasonic Impact Peening

**Figure 8:**
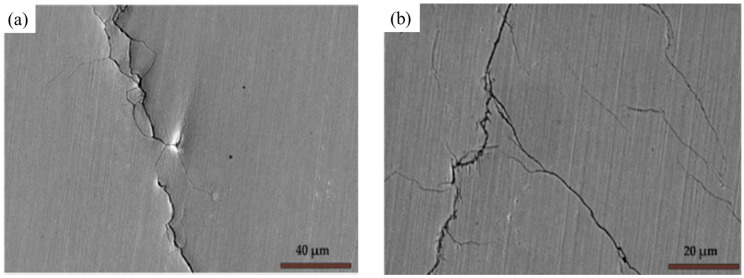
The SEM images of the crack morphology of CISCC cracks in the secondary electron mode. (**a**) U bend made from the as-received plate and (**b**) U bend made from the weld joint. Reproduced with permission from [17].

UIP offers unique advantages over CSP techniques. UIP possesses better controllability of process parameters, easy to operate, environmentally friendly, and flexible. In the UIP process, a cylindrical pin oscillates and strikes the substrate surface at an ultrasonic frequency [57]. Such a high frequency during UIP results in the substrate material to be mechanically peened [58]. The strain rate usually varies from (10^4^/s to 10^5^/s) [59]. During the SPD process, the substrate material develops surface hardening and strengthening effects. An SPD layer forms on the surface, containing nanograins and high-density dislocations. Scholars recommend UIP as a potential method to improve the strength and integrity of the weld joints [60]. Abdhullah et al. [27] showed superior enhancement in corrosion resistance of the SS 304 weld joint after UIP treatment. The enhanced corrosion resistance is attributed to RCS and microstructural changes induced during UIP. Ling and Ma [61] conducted SCC experiments on 304 weld joints in 42 wt.% boiling MgCl_2_. The authors observed that untreated weld joints failed within 23 h of testing. However, the weld that underwent UIP joint lasted 1000 h of testing without any visible cracks. Their results showed that the UIP process induced RCS and nanograins were achieved on the surface of the specimen, and they recommend UIP as an effective tool against CISCC issues in diverse industries. In a study, Kishore et al. [14] showed the enhanced CISCC resistance of the ASS 304L weld joint fabricated using GTAW for DSC application. The authors considered three U-bend specimens for their CISCC experiments in 42 wt.% boiling MgCl_2_. They observed that the U bend made from the as-received metal plate and weld joint failed in 15 and 12 h, respectively. The U bend specimens failed near the flange side. The scanning electron microscope (SEM) images of the cracks that appeared on the flange side are shown in Figure 8. These images shows the extensive crack propagation and disruptive nature of SCC. However, the specimens that underwent UIP withstand 300 h of testing without any visible cracks. The authors suggested that grain refinement and RCS provided superior CISCC resistance to the weld joints.

**Figure 9:**
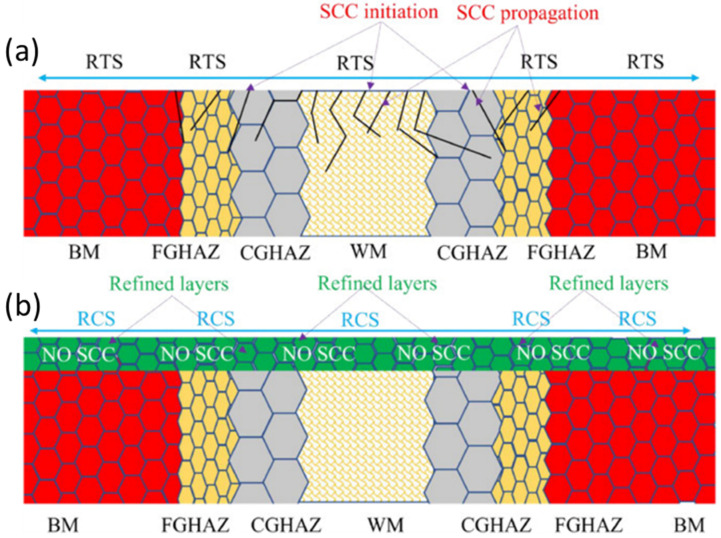
CISCC in ASS 304L weld joints (**a**) initiation and propagation, and (**b**) mitigation mechanism. Reproduced with permission from [17]. Copyright 2023, Elsevier.

The CISCC crack initiation and propagation are shown in Figure 9. The figure represents different zones including base material (BM), weld material (WM), fine-grain heat-affected zone (FGHAZ), and coarse-grain heat-affected zone (CGHAZ). The CISCC cracks initiate through different locations in the weld joint, such as the fusion zone (FZ) and HAZ (Figure 9a). The UIP treatment induced high RCS and grain refinement, preventing crack initiation and propagation (Figure 9b). The authors concluded that the grain refinement occurred via mechanical twins (MT) and twin–twin interaction. The MT separates the twin matrix lamellae into rhombic blocks, facilitating the formation of nanograins. The presence of nanograins can delay the crack propagation before it reaches the critical crack length [62].

In addition, the RCS on the surface extends the crack initiation time. The RCS in the subsurface region slows the crack propagation rate [63]. In this manner, the crack growth threshold can be reduced, which decreases the crack propagation rate. UIP can be considered an efficient and promising method to improve the CISCC resistance of the weld joints of DSCs. UIP modifies the weld geometry and introduces RCS and grain refinement. This extends the storage duration of the SNFs in DSCs without CISCC failure.

### 3.3. Ultrasonic Nanocrystal Surface Modification

Ultrasonic nanocrystal surface modification (UNSM) is another advanced SPD process that utilizes high-frequency ultrasonic vibrations combined with a static load to induce plastic deformation on the material surface [14,17]. The combined action of the static load and the impact force from the tooltip results in substantial plastic deformation on the material’s surface, introducing compressive residual stresses. This deformation generates a gradient microstructure with a nanograin surface layer, altering the mechanical properties within the affected region. Additionally, UNSM enhances surface quality by improving surface roughness and minimizing surface defects [48]. In the study carried out by Ye et al. [64], UNSM effectively transforms tensile residual stresses into high compressive residual stresses across the weld, heat-affected zone, and base metal. The process also induces surface nanocrystallization and significantly enhances hardness up to a depth of 350 μm. Corrosion testing in boiling MgCl_2_ solution shows that UNSM greatly improves the corrosion resistance of SS 304 welds.

### 3.4. Laser Shock Peening

**Figure 10:**
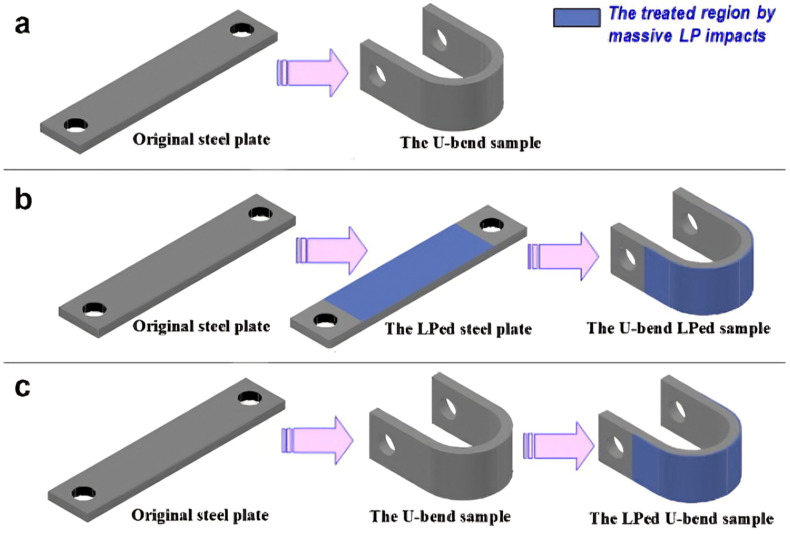
The three types of U bends are made from (**a**) flat plate, (**b**) LSPed flat plate and a further U bend, (**c**) U bend made from the flat plate and a further LSPed U bend. Reproduced with permission from [76]. Copyright 2012, Elsevier.

**Figure 11:**
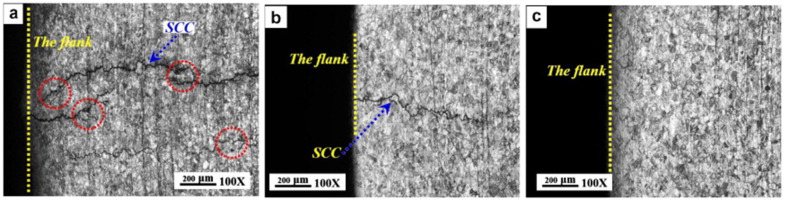
Optical microscopy images of the flange side of the U bend (**a**) showing CISCC failures in the first type of U bend (**b**) showing CISCC failures in the second type of U bend, and (**c**) no CISCC failure in the third type of U bend. Reprinted with permission from [76]. Copyright 2012, Elsevier.

During LSP, a coated substrate is exposed to a high-energy pulsed laser within a confinement medium [65]. The primary purpose of the coating is to absorb the laser’s intense energy, thereby preventing thermal ablation [18]. When a high-energy laser beam impacts the coated substrate within a confinement medium, it generates a laser-induced plasma that vaporizes the ablative material [66]. The confinement around the coated substrate restricts the plasma’s expansion, generating a high-pressure shock wave [67]. Consequently, the substrate material is subjected to an ultra-fast laser pulse, either nanosecond or femtosecond in duration. This laser pulse generates a high-pressure shock wave, reaching magnitudes in the gigapascal (GPa) range, and induces extreme strain rates of 10^5^/s to 10^6^/s along the surface during impact [68]. This process results in plastic deformation, the introduction of RCS, and the formation of a work-hardened, microstructurally refined layer [69,70,71]. LSP emerges as a highly effective surface modification technique, enhancing properties such as corrosion resistance, wear resistance, fatigue strength, and other critical mechanical characteristics [72,73,74,75]. The ablative coating is not a necessary requirement, and sometimes, the scholars conducted laser experiments without coating (LSPwC). Many scholarly works are available on LSP and LSPwC on stainless steel or nuclear applications. Lu et al. [76] performed massive LSP experiments on three types of U bend specimens made from ANSI 304L SS and conducted the SCC testing in 42 wt.% boiling MgCl_2_. These are demonstrated in Figure 10. For the first sample, a flat plate is converted into U bend specimens (Figure 10a). In the second case, the LSP was performed on the flat specimen, and U bend specimens were made (Figure 10b, represented as LSPed). In the third case, a U bend specimen is made from a flat plate, then LSP is carried out (Figure 10c). In all these cases, the type of residual stress affects the CISCC resistance. The optical microscopy images of the failed U bend is shown in Figure 11. The first type U bend specimen failed in 16 h (Figure 11a). This is due to high RTS in the U bend, causing easy crack initiation and propagation. The cracks are marked in red circle. The second type U bend, failed at approximately 110 h (Figure 11b). This is because of the grain refinement and RCS resulting from LSP. However, when a U bend is made from this flat plate, RCS is converted into RTS, which is low in magnitude compared to the first type of U bend.

In the third type of U bend, the presence of RCS and grain refinement raised the failure time to a great extent. The authors did not observe failure for up to 300 h of testing (Figure 11c). The RCS raise the crack growth threshold and reduce the rate of crack propagation [72]. This recommends that LSP be a feasible post-processing technique to limit the CISCC issues in DSCs.

Lu et al. [77] performed LSP on SS 304 and reported the increase in corrosion resistance with increasing pulse energy. This improvement was attributed to the extensive LSP impacts, which widened the shear-lip region, leading to refined and uniformed dimples, and expanding the necking region.

Sundar et al. [78] did machining on SS 304L and induced RTS before conducting CISCC studies. Further, authors conducted LSP and oblique LSP, and revealed that both techniques could enhance the CISCC resistance. Their studies demonstrated that surface modification techniques were essential when machined components were working in chloride environments. Table 1 summarizes the various peening techniques on SS materials and weld joints and corresponding observations.

**Figure 12:**
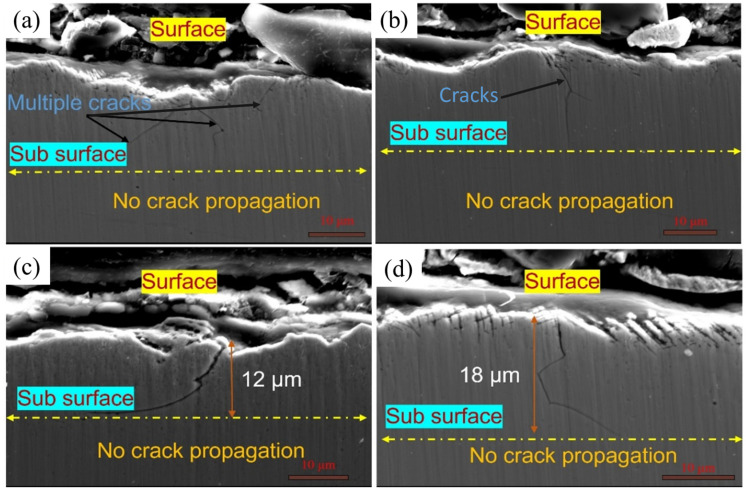
SEM of the failed SCC tested samples showing cracks originating from the surface and not propagated into the bulk (**a**,**b**) cracks at two locations near the flange side, (**c**,**d**) of different dimensions. Reproduced with permission from [39].

Recently, most works focus on the LSPwC. The LSPwC is a thermo–mechanical coupling process that provides superior surface strengthening and surface hardening compared to LSP [80]. In LSPwC, there is a competition of thermo-mechanical effects during each pulse. However, it is still debated how LSPwC influences the resistance to CISCC. Lu et al. [81] observed RCS on the peened surface of SS 304L following LSPwC, a finding corroborated by Prabhakaran et al. [82]. In contrast, experiments conducted by Nataraj et al. [83] on SS 304L using LSPwC revealed RTS on the surface, with RCS confined to the subsurface region. This phenomenon is further elaborated by Praveenkumar et al. [84]. Therefore, there is a growing need to understand how the RTS on the surface and RCS in the subsurface interact with SCC. To understand the effect of the type of stress on CISCC resistance on the SS 304L that underwent GTAW, LSPwC experiments were conducted [39]. Their studies revealed the occurrence of RTS on the surface of the weld joints. During LSPwC, the interaction between the laser and the substrate results in the formation of an oxide layer and the development of RTS on the surface. These surface RTS are attributed to the effects of laser ablation. Following the LSPwC process, the treated region undergoes cooling and contraction, with the surrounding material constraining this contraction. As a result, RTS are developed on the surface. The RTS was present only up to a few µm, and the material that underwent LSPwC had the RCS to appear below 25 µm 50 µm. The RCS on the subsurface region arise from the synergistic mechanical effects of shock wave propagation, which induces non-uniform plastic strain and minimizes laser ablation [85]. The SCC studies on the U bends subjected to LSPwC showed cracks after approximately 118 h of testing. However, the U bends were tested up to 300 h to see if the subsurface RCS is capable enough to prevent the crack propagation into the bulk of the specimen. Multi-branch cracks were observed whose length varies from 10 µm to 20 µm. However, these cracks were not able to propagate into the bulk of the specimen. This is due to high-magnitude RCS in the subsurface region and grain refinement. The SEM images of the crack and its propagation are shown in Figure 12a,b. Cracks of different dimensions are shown in Figure 12c,d.

Recently, laser shock surface patterning (LSSP) has emerged as a promising technique for enhancing surface properties [86]. John et al. [87] studied SCC failures in ASS weld joints using LSSP. LSSP was applied with varying intensities to the fusion zone, heat-affected zone, and base material. SCC tests on U-bend specimens in boiling MgCl_2_ showed that LSSP induced strain-induced martensitic transformation (α′-phase), RCS, and grain refinement, improving surface hardness and reducing roughness and α′-phase volume. LSSP-treated specimens exhibited enhanced SCC resistance compared to unpeened specimens, attributed to RCS in the subsurface and minimal impact of surface roughness and α′-phase.

**Figure 13:**
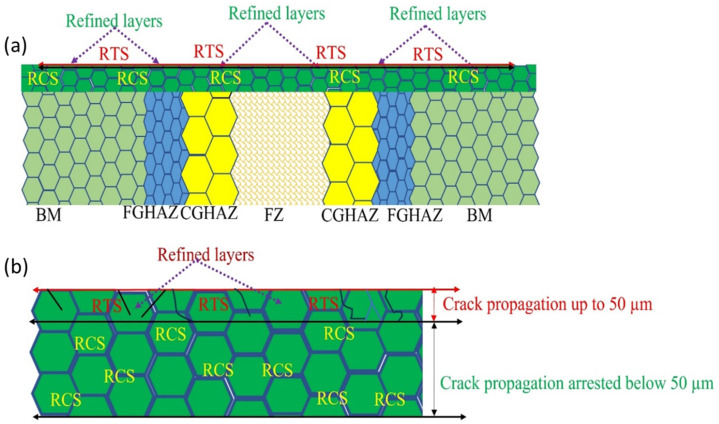
Mitigation mechanism of CISCC (**a**) showing RTS on the surface and RCS in the subsurface regions, and (**b**) magnified view of the refined layer. Reproduced with permission from [39].

In conclusion, the RTS induced on the weld joint surface by the thermal effects of LSPwC do not adversely affect the SCC performance of the weld joint. This is because the mechanical effects of LSPwC generate a work-hardened layer beneath the surface, enhancing the material’s mechanical properties and contributing to improved overall performance. Irizalp et al. [88] also reported similar effect during LSPwC on ASS. The crack inhibition mechanism after LSPwC on the SS 304L weld joint is indicated in Figure 13a,b.

These studies illustrate that LSPwC is an effective surface enhancement method that can prevent the CISCC failures of DSC weld joints.

## 4. Evaluation Techniques for SCC

SCC in austenitic stainless steels is a major concern, especially in harsh environments like those with chlorides, high temperatures, or other corrosive conditions. To understand how these materials behave and resist SCC, testing methods are designed to mimic real-world service conditions as closely as possible. This section briefly discuss various testing methods, specifically, the three methods—constant load, constant strain, and the slow strain rate technique (SSRT)—to evaluate the susceptibility of a material to SCC.

### 4.1. The Constant Load Test

**Figure 14:**
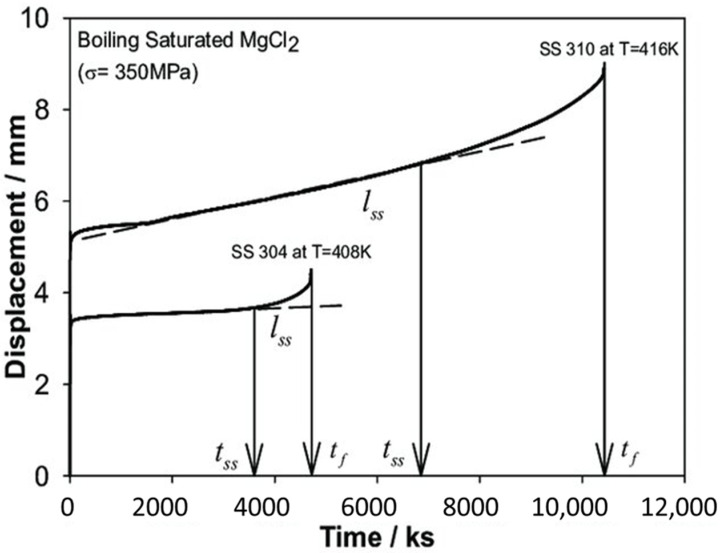
Corrosion elongation curve for 304 steel and 310 steel under constant load test in boiling saturated MgCl2 [91].

A constant load test for SCC involves the application of a constant tensile load to a specimen while immersing the specimen in a corrosive environment. The testing is carried out to allow for the cracks to initiate and propagate. By monitoring the time to failure at the specific load, the material’s susceptibility to SCC is evaluated. In the constant load method, both the time to failure and elongation can be precisely measured; however, these tests require a significant amount of time to assess the susceptibility to SCC. The constant load method has been widely employed to study the SCC behavior of ASSs, focusing on factors such as applied stress, anion concentration, test temperature, and pH. It was observed that the steady-state elongation rate (I_ss_) derived from the corrosion elongation curve serves as a key parameter for predicting the time to failure [89,90]. The steady-state elongation rate is the slope of the linear portion of the curve representing the stable crack propagation phase. This parameter is essential for comparing the SCC behavior of various materials or under different environmental conditions. A higher steady-state elongation rate signifies faster crack propagation. The corrosion elongation curve for 304 steel and 310 steel at 408 K and 416 K, respectively, under a constant stress condition (σ = 350 MPa) in boiling saturated MgCl_2_ is shown in Figure 14. The three parameters that can be obtained from the curve is Time to failure (t_f_), steady-state elongation rate (I_ss_), and transition time (t_s_), where t_s_ is the time at when the elongation curve begin to deviate from a linear increase [5].

### 4.2. The Constant Strain Method

The constant strain test is commonly used to assess the susceptibility of materials to SCC under constant strain. A typical method within this approach is the U-bend test, where a specimen is bent into a U shape and exposed to a corrosive environment while maintaining a constant strain. The applied bending creates stress on the specimen, and the test helps evaluate how the material responds to these conditions. As the test progresses, any cracking that occurs is monitored, providing important information about the material’s vulnerability to SCC in various environments. The test measures the time it takes for cracks to initiate and propagate on the specimen, indicating the material’s resistance to SCC. The U-bend specimen is typically a rectangular strip that is bent 180° around a specified radius, and this constant strain is maintained throughout the stress-corrosion testing process, based on ASTM G30 standard [92].

### 4.3. Slow Strain Rate Testing

Slow strain rate testing (SSRT) is a tensile test conducted on a standard smooth tensile specimen, where the specimen is gradually stretched at a constant strain rate until fracture occurs. The crosshead speed is regulated to achieve strain rates, typically ranging from 10^−4^ to 10^−7^ s^−1^ [93]. The critical strain rate is the strain rate at which stress corrosion cracking occurs most rapidly in a given environment [94]. While the slow strain rate test (SSRT) is a useful method for assessing the SCC proneness of materials in corrosive environments, it is essential to compare parameters such as time to failure, maximum stress, and strain, derived from the stress–strain curve in a corrosive environment, with those obtained in an inert environment for accurate evaluation.

## 5. Factors Affecting SCC Resistance

SCC involving the interplay between mechanical stress, corrosive environment, and material susceptibility. Such localized corrosion forms can lead to sudden, catastrophic failures in structures and components at relatively low levels of stress; which is a major concern for important industries like nuclear energy, aerospace, marine, and chemical processing. Whereas general corrosion is seldom aggressive in materials like ASSs, SCC may be very serious under conditions of aggressive environments containing chlorides. Resistance to SCC depends on material composition and microstructure, the type of stress involved, and magnitude. Environmental conditions such as temperature, pH, and the concentration of corrosive agents are also very crucial in SCC initiation and propagation. Additionally, manufacturing processes, including welding and surface treatments, can introduce residual stresses or microstructural changes that affect SCC behavior.

SCC can occur in certain pure metals under specific environmental conditions, but alloys are generally more susceptible due to their complex composition and microstructure. Key factors such as alloy composition, microstructure, and microchemistry—particularly near grain boundaries—significantly influence SCC resistance. Alloy composition and microstructure also determine material strength, and for many materials, especially steels, there is a notable trend of increased SCC susceptibility with higher strength levels [95,96].

Grain morphology and size play a critical role in SCC behavior. Rolled, forged, or extruded materials often exhibit elongated grain structures, which can reduce SCC resistance when stressed in short-traverse crack-plane orientations. Additionally, the distribution of grain-boundary misorientaions, or texture, is crucial. Special boundaries, such as low-angle and coincidence-site boundaries, exhibit much greater resistance to SCC compared to general high-angle grain boundaries [95,97].

The composition of the solution, including ionic species and their concentrations, pH, dissolved oxygen content, temperature, and electrode potential, significantly impacts SCC resistance. In some cases, even trace amounts of certain ions can dramatically influence susceptibility. For instance, the susceptibility of γ-stainless steels increases significantly when chloride ion concentrations exceed a specific threshold (measured in ppm), which varies depending on other environmental factors [98].

The surface condition of a material significantly influences its resistance to SCC. Surface roughness can create stress concentration points that are prone to crack initiation, while smoother surfaces distribute stress more evenly, reducing SCC susceptibility. Surface defects, such as scratches, pits, or inclusions, often introduced during manufacturing processes like machining or welding, can serve as initiation sites for cracks. Additionally, residual stresses from manufacturing, such as tensile stresses from welding, can promote SCC, whereas compressive stresses can mitigate it. Surface treatments like polishing, shot peening, or applying protective coatings can enhance SCC resistance by reducing roughness, eliminating defects, and introducing beneficial compressive stresses [99]. 

Understanding these factors is important for engineers and materials scientists to design strategies to increase the resistance of SCC. These may include selecting high-resistance materials, improving welding techniques, applying protective coatings, or modifying operating conditions.

## 6. Recent Advances

The peening parameters should be severe to obtain superior properties such as fine grain size (nano grains) and in-depth RCS. When choosing severe parameters for superior surface mechanical properties, the surface roughness of the treated specimen increases. However, the increase in surface roughness during the process is detrimental to the CISCC resistance [46]. The higher surface roughness of the substrate promotes an easy pit initiation on the weld joints of DSCs. Surface roughness directly relates to CISCC resistance of DSC weld joints. Hence, it is crucial to develop techniques capable enough to minimize the surface roughness of the treated surface along with the same or improved benefits compared to peening techniques. One such newly developed method is the ultrasonic surface rolling process (USRP)**.** This was a prominent development in the surface engineering field. Another development is the introduction of advanced repair techniques named high-pressure cold spray (HPCS) deposition. HPCS was widely employed in diverse industries as a prominent repair method. In addition to this, new methods of joining DSCs were developed, which is advanced hybrid laser and arc welding (HLAW). HLAW techniques could develop defect-free weldments with minimum RTS distribution in the FZ and HAZ. The following section illustrates the development of these techniques and discusses the unique abilities inherent to these techniques.

### 6.1. The Ultrasonic Surface Rolling Process

**Figure 15:**
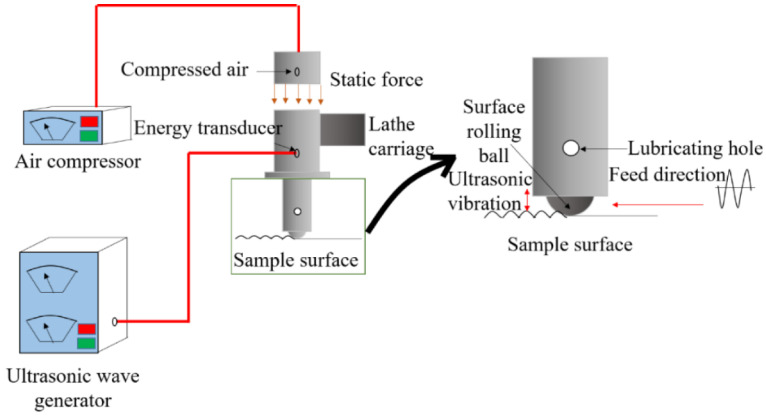
Working principle of USRP. Reproduced with permission from [100]. Copyright 2022, MDPI.

USRP is a novel SPD technique that combines the benefits of UIP and deep rolling (DR) [100]. USRP techniques were developed in 2008 to obtain superior surface properties at a reduced surface roughness [101]. The schematic of the USRP is illustrated in Figure 15. The USRP consists of an ultrasonic wave generator, compressor, and tooltip. The tooltip is associated with a lathe carriage [102]. The tooltip is round, and a static force is applied to the tooltip that performs the rolling operation. The tooltip vibrates at ultrasonic frequency, and static force is applied through compressed air. The repeated impact of the tooltip at such a high frequency, along with the static force, produces both elastic and plastic deformation on the substrate [100]. After the USRP, the elastic deformation recovers, and plastic deformation significantly reduces the surface roughness of the treated specimen. The oil through the lubricating hole in the tooltip reduces the friction between the tooltip and substrate material. In addition, this acts as a coolant, improves the treated surface appearance, and provides superior dimensional tolerance [103].

**Figure 16:**
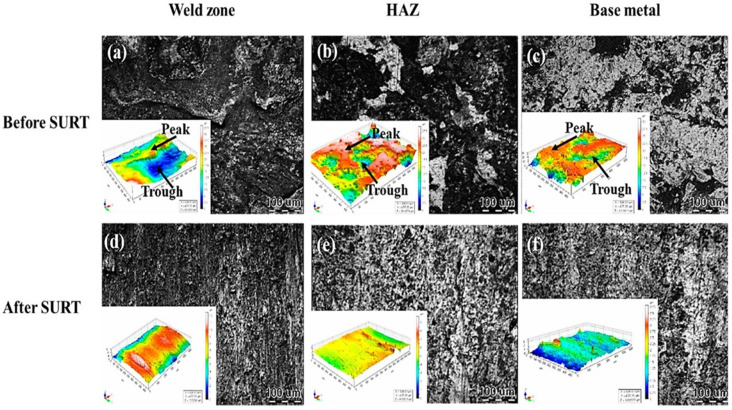
Surface morphology and surface roughness of the untreated (**a**) weld zone, (**b**) HAZ, (**c**) and base material before SURT and (**d**) weld zone, (**e**) HAZ, (**f**) base material after SURT. Reprinted with permission from [110]. Copyright 2020, MDPI.

Researchers performed USRP experiments on different engineering materials, such as steel grades, titanium alloys, aluminum alloys, and magnesium alloys [104,105,106,107]. The authors summarized that USRP can enhance corrosion resistance, fatigue, wear resistance, and surface hardness at a lower surface roughness [108,109]. Liu et al. [110] performed surface ultrasonic rolling treatment (SURT) treatment, another name for the USRP on the T40003 ferritic steel weld joint, and they observed a reduction in surface roughness in the weld zone, HAZ, and base metal. The initial surface roughness and morphology of the weld zone, HAZ, and base material are 1.06 μm, 2.18 μm, and 2.160 μm (Figure 16a–c). However, SURT the roughness was reduced to 0.320 μm, 0.156 μm, and 0.227 μm, respectively (Figure 16d–f). The surface roughness reduction reduces the stress concentration, thereby improving the CISCC resistance [111].

### 6.2. High-Pressure Cold Spray

**Figure 17:**
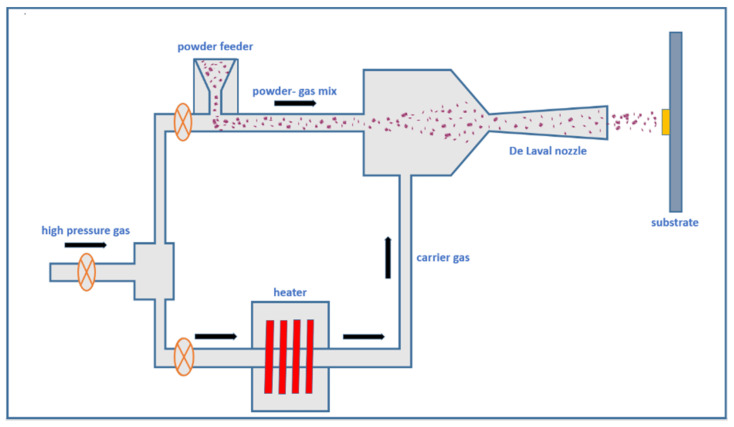
Working principle of HPCS. Reproduced with permission from [114]. Copyright MDPI, 2022.

The HPCS is an advanced method of powder particle deposition that is a solid state coating process with minimal heat input [112]. The HPCS can be used to restore dimensions, repair damages, and manufacture near-net shape components [113,114]. The HPCS system is shown in Figure 17. During HPCS, gases such as nitrogen or helium under high pressure (up to 1000 psi) are preheated and propelled through a converging-diverging De-Laval nozzle. When the high-pressure preheated gas flows through the nozzle, it converts enthalpy into kinetic energy, and expansion occurs, accelerating the gas to a supersonic speed. The powder to be coated on the substrate is axially fed into the gas stream before it reaches the nozzle. The powder particle accelerates and impacts the substrate. This occurs at high velocity and pressure, and the kinetic energy of the powder particle is used for deposition rather than the thermal energy [115,116]. Upon impingement on the substrate, the powder forms dense and strong adherent coatings. This process induces a high strain rate during the plastic deformation of powder particles, and involves the adiabatic shear instability to occur at the inter-particle and particle/substrate interfaces [117].

**Figure 18:**
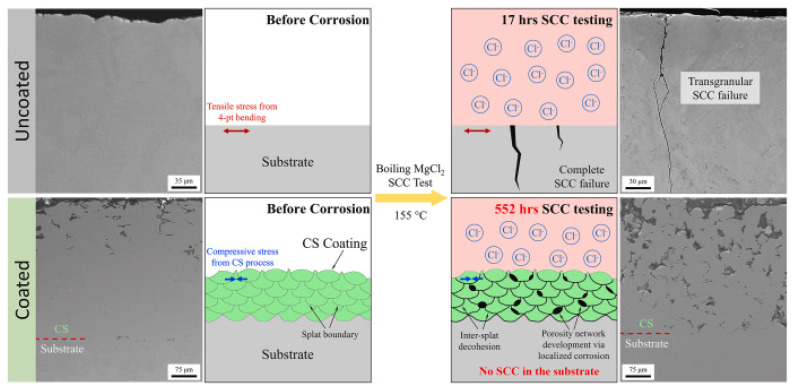
The coated and uncoated substrate before corrosion (left side) and the coated and uncoated substrate after corrosion. Reproduced with permission from [121]. Copyright Elsevier, 2022.

**Figure 19:**
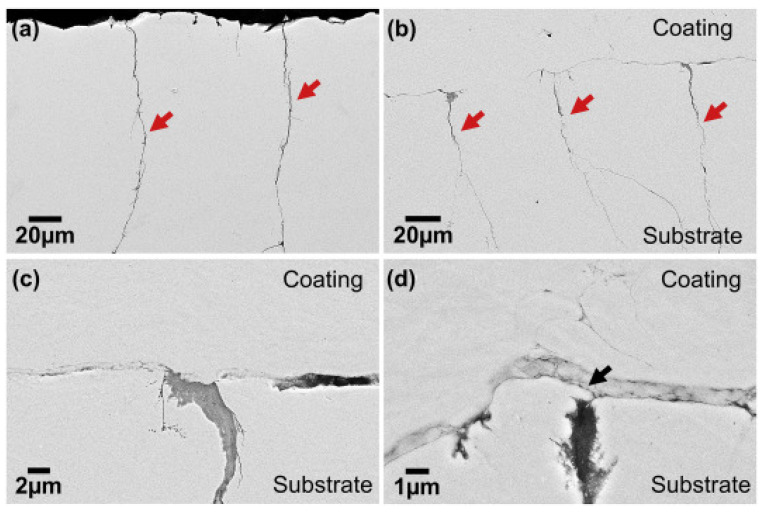
SEM of (**a**) CISCC in SS 304 L, (**b**) HPCS coated substrate, (**c**) high magnification at the coating/CISCC interface overlay coating at the crack opening, and (**d**) sealing the crack opening by substrate deformation. Reproduced with permission from [122]. Copyright Elsevier, 2015.

The nature of the deformation and bonding mechanism, such as metallurgical bonding and mechanical interlocking, plays a predominant role in the coating [118,119]. Proper selection of HPCS process parameters is essential to deposit a high-quality coating on the weld joints. Despite the fact that the HPCS is not a straightforward peening technique, the supersonic particle deposition of powder causes a peening effect on the substrate, causing severe plastic deformation on the welded joint [120]. This can enhance DSCs’ corrosion resistance, wear resistance, hardness, surface characteristics, and longevity. The effect of RCS prevents the potential CISCC issues in the nuclear canisters. Recently, Qu et al. [121] conducted HPCS deposition of SS 304L powders on SS 304L joints that underwent GTAW and demonstrated enhanced CISCC resistance of coated substrate in boiling MgCl_2_. The coated specimen tested for up to 552 h showed no signs of CISCC in the substrate. However, the uncoated substrate failed at approximately 17 h. The authors suggested that peening effects during HPCS deposition induced RCS that enhanced the CISCC resistance of the weld joints. The proposed CISCC mechanism is shown in Figure 18. The authors performed a four-point bend test and immersed the specimen in boiling MgCl_2_. The SEM of the uncoated and coated substrate before SCC testing is shown in Figure 19 (left side). After the immersion in boiling MgCl_2,_ the uncoated substrate failed by transgranular CISCC.

The coated substrate showed signs of localized crevice corrosion at the intersplat boundaries shown in Figure 18 (right side). The decohesion of intersplat boundaries reduces the RCS in the coating. Even if the corrosive species reaches the substrate, it cannot cause CISCC. This is because of the presence of RCS in the substrate. This demonstrates the unique ability of HPCS against CISCC. Yeom et al. [122] conducted HPCS experiments on SS 304L for DSC application. In their experiments, the authors used SS 304L powders with a 20–45 μm particle size. Both nitrogen and helium mixtures were used as carrier gas, and they were preheated to 550 °C. The authors reported the coating had superior adhesion strength on to the substrate. A gradient variation in particle size was found along the depth direction of the coating. Figure 19 shows how the CISCC in the SS 304L is prevented during HPCS deposition. The cracks (marked using arrows) appeared on the SS 304L at approximately 41 h (Figure 19a), and the crack size was less than 10 μm. The SS 304L deposits on the substrate are shown in (Figure 19b).

The obtained coating was 700 μm thick. There are two ways in which HPCS deposition helps to prevent the CISCC in the substrate. In the first method, the deposited coating sealed the crack in the substrate. Since the powder particle size was higher than the crack opening, they could fully cover the CISCC in the substrate (Figure 19c). Secondly, the SPD in the substrate during HPCS caused the crack sealing. The SPD occurred in normal and lateral direction to the substrate. Thus, it completely closed the crack opening (Figure 19d). These studies demonstrate that HPCS is a prominent and effective repair technique against CISCC.

### 6.3. Hybrid Laser and Arc Welding

**Figure 20:**
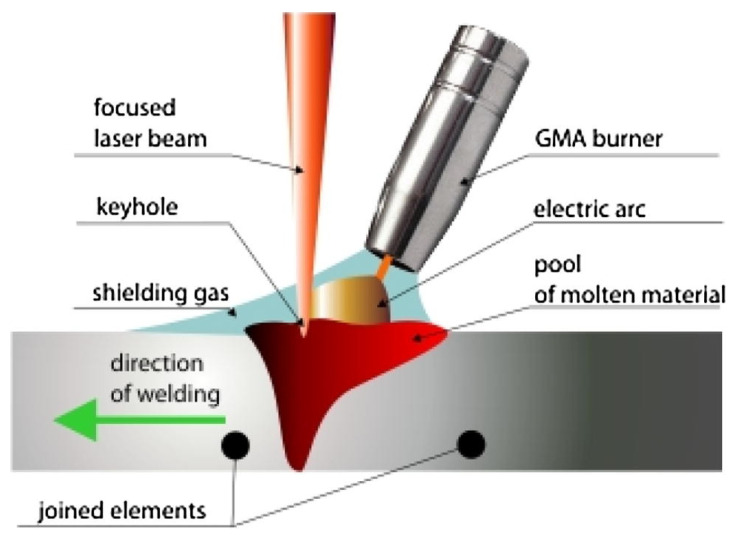
The HLAW process. Reproduced with permission from [123]. Copyright Elsevier, 2018.

Specific applications demand superior CISCC resistance of weld joints without any post-processing. In this situation, adopting a new welding technique is of great interest. HLAW technique can efficiently handle this situation. HLAW combines GMAW and laser beam welding (LBW) techniques to manufacture the weld joints for DSC applications. This process can prevent potential setbacks associated with conventional GMAW [123]. LBW generates a deep and narrow penetration and can produce single-pass full penetration welds in ASS plates as thick as 1 in. HLAW could develop full penetration weld in one pass at high speeds and accommodate more significant variations in joint fit-up than standard LBW. The concentrated laser energy source and fast travel speed of HLAW provide significantly lower heat input and a smaller FZ and HAZ than GMAW [124,125]. The HLAW process enhances the gap-bridging capability and decreases the steepness in the thermal gradient. This results in substantially lower RTS levels and distortion than the conventional GMAW [126]. Another advantage of HLAW is that fusion zone microstructure can be tailored to obtain desired mechanical properties [127]. The schematic of HLAW is shown in Figure 20. The HLAW process is associated with many parameters; hence, an appropriate selection of parameters is vital to fabricating weld joints with minimum defects and residual stress distribution [128]. Fu et al. [129] performed HLAW experiments on the SUS30L-MT steels and conducted SSRT tests on the base material and weld joints. The authors reported low CISCC susceptibility in HLAW joints compared to a high value for the base material. The weld joint showed 2.56-fold lower CISCC susceptibility than the base material. The authors summarized that it is due to the cathodic protection of the austenite originating from the corroding ferrite-austenite interface.

Lowering residual weld stress reduces the risk of CISCC, a critical issue in the nuclear industry. HLAW is widely used throughout many industries for welding thick structures, and it is being advanced by the Department of Energy (DOE) for nuclear vessels [130]. The weld joints made using HLAW are expected to have superior pitting corrosion and CISCC resistance than conventional GMAW. The improved CISCC resistance and longevity of DSCs can save millions of dollars in repair and rework costs. The primary expected outcome of HLAW will be increased safety and security against CISCC in DSCs.

## 7. Conclusions

This review article provides an extensive discussion on peening techniques of surface modification for enhancing the CISCC resistance of DSC weld joints used for storing SNFs. Peening techniques are considered robust, economical, and industrially viable top-down approaches to enhance the integrity of surface mechanical properties. This review presents a thorough overview of the state-of-the-art peening techniques and their impact on the CISCC resistance of DSC weld joints. This review summarizes key peening processes, including CSP, UIP, and LSP, and their effects on enhancing CISCC resistance. Peening techniques vary in effectiveness, with advanced methods like LSP and UNSM standing out for their ability to enhance material properties. LSP provides deep compressive stress layers and minimal surface damage, while UNSM offers a cost-effective balance of improved hardness, wear, and corrosion resistance. Shot peening remains a widely used, budget-friendly option but is limited by shallower effects and potential surface defects. Despite their benefits, these techniques come with some challenges. For instance, traditional methods like shot peening can sometimes lead to surface defects or contamination. Similarly, conventional ultrasonic peening might not achieve the deeper compressive stress layers or the level of nanocrystallization that more advanced techniques offer. Additionally, the high cost and technical complexity of methods like LSP and UNSM can make them less accessible for widespread use. Among the various techniques, LSP is considered the most powerful due to its ability to create deep compressive stress layers with minimal surface damage.

This review also explores testing methods used to evaluate material susceptibility to SCC and examines various factors influencing SCC resistance. Additionally, advancements in surface modification techniques, such as USRP and LSSP, are discussed, with particular emphasis on the importance of surface roughness. Innovative repair methods like HPCS and advanced welding techniques such as HLAW are also highlighted. This review offers valuable insights into the role of peening and surface modification techniques in improving the CISCC resistance of DSC weld joints, particularly for SNF storage applications.

## Figures and Tables

**Table 1:** Peening techniques on SS materials and weld joints.

Material	Peening Techniques	Type of SCC Test	Observations	Reference
ASS 304L weld	SP	SSRT	An increase in SP pressure increases the martensite content, grain refinement, and SPD Higher shot peening pressure increases the stress corrosion sensitivity index The optimum SP pressure was identified to be 0.4 MPa	[45]
SS 304	SP	Boiling MgCl_2_	RTS changed to RCS SPD layer of 0.35 mm observed RCS and grain refinement prevented the CISCC The RCS prevented the rupturing of the oxide film	[50]
SS 304 weld	MSP	Salt spray containing 10% NaCl	Austenite to martensite transformation occurred A high martensite fraction was observed on the fusion zone of the weld, and more pits were observed Nano grain formation and RCS observed CISCC prevented	[51]
SS 316 weld	MSP	Salt spray containing 10% NaCl	Austenite to martensite transformation occurred Nano grain formation and RCS observed CISCC prevented	[51]
SS 304 weld	UIP	Cyclic voltammetry	The weld joint has a high corrosion rate before peening Increased corrosion resistance of peened weld joint Fatigue properties improved	[27]
SS 304 weld	UIP	Boiling MgCl_2_	Untreated weld joint failed in 23 h Weld joint that underwent UIP failed in 1000 h of testing Ultrafine grain formation on the top surface	[61]
ASS 304L weld	UIP	Boiling MgCl_2_	Nano grain formation and in-depth RCS induced by UIP Untreated U bend specimen failed at approximately 14 h U bend specimens from the weld joint that underwent UIP withstand 300 h of testing	[14]
SS 304	UNSM	Boiling MgCl_2_	UNSM converts RTS into high RCS in the weld, heat-affected zone, and base metal regions Enhanced surface hardness up to 350 μm Nanocrystallization in the processed surface Improved corrosion resistance	[64]
ANSI SS 304L	LSP	Boiling MgCl_2_	U bend specimens were able to withstand 300 h of testing Improved CISCC resistance compared to U bends made from flat, and U bends made from flat plate subjected to LSP Nano grain formation and MT observed on the surface LSP induced high RCS on the surface	[76]
SS 304 weld	LSP	Boiling MgCl_2_	Improved surface hardness, grain refinement, and RCS introduction Cracks initiated on specimens treated with LSP are higher compared to untreated specimen The electrochemical corrosion resistance of the specimens that underwent LSP improved	[79]
AISI SS 304	LSP	0.598 mol/L NaCl solution	SCC and electrochemical corrosion resistance increased with increasing pulse energy Enhanced corrosion resistance is attributed to surface layer with RCS and refined grains	[77]

## Abbreviations

The following abbreviations are used in this manuscript:

ASS	Austenitic Stainless Steel
DSCs	Dry Storage Canisters
SNF	Spent Nuclear Fuel
CISCC	Chloride-Induced Stress Corrosion Cracking
SP	Shot Peening
CSP	Conventional Shot Peening
UIP	Ultrasonic Impact Peening
LSP	Laser Shock Peening
RTS	Residual Tensile Stress
RCS	Residual Compressive Stress
GMAW	Gas Metal Arc Welding
HAZ	Heat-Affected Zone
SPD	Severe Plastic Deformation
MSP	Micro Shot Peening
SSP	Severe Shot Peening
UNSM	Ultrasonic Nanocrystal Surface Modification
SSRTT	Slow Strain Rate Tensile Test
FGHAZ	Fine-Grain Heat-Affected Zone
CGHAZ	Coarse-Grain Heat-Affected Zone
BM	Base Material
WM	Weld Material
FZ	Fusion Zone
MT	Mechanical Twins
LSSP	Laser Shock Surface Patterning
HPCS	High-Pressure Cold Spray
HLAW	Hybrid Laser Arc Welding
USRP	Ultrasonic Surface Rolling Process
